# Piperlongumine Blocks JAK2-STAT3 to Inhibit Collagen-Induced Platelet Reactivity Independent of Reactive Oxygen Species^†^

**DOI:** 10.1371/journal.pone.0143964

**Published:** 2015-12-08

**Authors:** Hengjie Yuan, Katie L. Houck, Ye Tian, Uddalak Bharadwaj, Ken Hull, Zhou Zhou, Mingzhao Zhou, Xiaoping Wu, David J. Tweardy, Daniel Romo, Xiaoyun Fu, Yanjun Zhang, Jianning Zhang, Jing-fei Dong

**Affiliations:** 1 Bloodworks Northwest Research Institute, Seattle, Washington, United States of America; 2 Tianjin Neurological Institute, General Hospital, Tianjin Medical University, Tianjin, China; 3 Division of Infectious Disease, Department of Medicine, Baylor College of Medicine, Houston, Texas, United States of America; 4 The Natural Products LINCHPIN Laboratory and Department of Chemistry, Texas A & M University, College Station, Texas, United States of America; 5 Medicine Division, Imperial College London, London, United Kingdom; 6 Division of Hematology, Department of Medicine, University of Washington, School of Medicine, Seattle, Washington, United States of America; University of Kentucky, UNITED STATES

## Abstract

**Background:**

Piperlongumine (PL) is a compound isolated from the *piper longum* plant. It possesses anti-cancer activities through blocking the transcription factor STAT3 and by inducing reactive oxygen species (ROS) in cancer, but not normal cells. It also inhibits platelet aggregation induced by collagen, but the underlying mechanism is not known.

**Objective:**

We conducted *in vitro* experiments to test the hypothesis that PL regulates a non-transcriptional activity of STAT3 to specifically reduce the reactivity of human platelets to collagen.

**Results:**

PL dose-dependently blocked collagen-induced platelet aggregation, calcium influx, CD62p expression and thrombus formation on collagen with a maximal inhibition at 100 μM. It reduced platelet microvesiculation induced by collagen. PL blocked the activation of JAK2 and STAT3 in collagen-stimulated platelets. This inhibitory effect was significantly reduced in platelets pretreated with a STAT3 inhibitor. Although PL induced ROS production in platelets; quenching ROS using excessive reducing agents: 20 μM GSH and 0.5 mM L-Cysteine, did not block the inhibitory effects. The NADPH oxidase inhibitor Apocynin also had no effect.

**Conclusions:**

PL inhibited collagen-induced platelet reactivity by targeting the JAK2-STAT3 pathway. We also provide experimental evidence that PL and collagen induce different oxidants that have differential effects on platelets. Studying these differential effects may uncover new mechanisms of regulating platelet functions by oxidants in redox signals.

## Introduction

The long pepper (*Piper longum*) is a member of the Piper genus known to possess anti-allergenic, anti-inflammatory, and liver protecting properties [1;2]. The fruit of *P*. *longum* has more than 600 known active compounds; among them is piperlongumine (PL, 5,6-dihydro-1-[1-oxo-3-(3,4,5-trimethoxyphenyl]-2(1H) pyridinone). PL, related compounds, and chemically synthesized analogs are reported to have anti-cancer [2;3], gastric protective [4], anti-microbial [5;6], adipogeneic [7;8], and anti-atherosclerotic activities [9]. PL executes these diverse activities, in part, by targeting protein synthesis at the transcriptional and post-transcriptional levels [3;10–12]. Raj L, *et al* [11] reported that PL selectively induces the death of cancer or transformed cells, while preserving the viability of normal cells *in vitro* and *in vivo*. This targeted activity relies on PL’s ability to increase intracellular reactive oxygen species (ROS), especially hydrogen peroxide and nitric oxide [11]. PL does so by binding and down-regulating activities of the enzymes glutathione S-transferase pi 1 (GSTP1) and carbonyl reductase 1 (CBR1)[11], resulting in a redox potential that is significantly tilted towards oxidation[3;11].

Paradoxically, PL was also reported to inhibit platelet activation, secretion, and aggregation induced by collagen, arachidonic acid, thromboxane, and platelet activating factor [13–15]. However, the known mechanisms involved in the inhibition of cancer cells do not support PL’s actions in platelets. First, blocking gene transcription is unlikely to play a significant role in the anti-platelet activity of PL because platelets are anucleated cells with a limited capacity for protein synthesis. Furthermore, the inhibitory effect was detected within minutes after PL treatment, insufficient for significant transcription and/or translation activities to occur. Second, ROS is known to activate platelets and promote thrombosis [16;17]. Cross-linking GP VI by collagen stimulates platelets to produce ROS [18]. These conflicting data lead to a critical question of whether PL induces these diverse phenotypic changes in platelets and cancer cells by targeting different molecules or by acting on a single molecule that is active both transcriptionally and non-transcriptionally. A target of PL specifically for collagen-induced platelet activation and aggregation that emerged from our recent study is the transcription factor STAT3 [19].

STAT3 is a member of a family of seven closely related proteins and serves as a key signaling protein in the IL-6-induced acute phase reaction pathway [20]. In nucleated cells, IL-6 binds to the IL-6Rα-gp130 receptor complex, oligomerizing receptors to activate receptor-associated tyrosine kinases. These activated kinases phosphorylate tyrosine residues to create a docking site for STAT3 via its Src-homology 2 (SH2) domain [21], leading to the phosphorylation of STAT3 at residue Y705 and the formation of a tail-to-tail homo-dimer that accumulates in the nucleus to regulate the transcription of targeted genes. We have recently uncovered a non-transcriptional role of STAT3 in a cross-talk between inflammatory IL-6-STAT3 and hemostatic/thrombotic collagen-GP VI signals that renders platelets hyper-reactive in conditions of inflammation through a trans-signaling mechanism [22].

These published reports have led us to hypothesize that PL regulates a non-transcriptional activity of STAT3 to reduce platelet reactivity to collagen. Here, we present data from *in vitro* experiments on human platelets using PL and synthesized derivatives to support this hypothesis. This study was not designed to study the pharmacokinetics of PL, but to identify PL’s molecular target(s) in regulating collagen-induced platelet activity and to explore roles of oxidative stress in platelet reactivity.

## Materials and Methods

### Reagents

Commercial reagents used in the study included: PL (Cayman Chemical Co., Ann Arbor, MI), the STAT3 inhibitor STA21 (Sigma Aldrich, St. Louis, MO), the JAK2-inhbitor AG490 (InvivoGen, San Diego, CA), the Syk inhibitor SykII (Merck Millipore, Billerica, MA), human recombinant IL-6 (R & D Systems, Minneapolis, MN), the extracellular domain of human IL-6 receptor-α (R & D Systems), Actinomycin (Sigma Aldrich), apocynin (Abcam Biochemicals. Cambridge, MA), free glutathione (GSH, Sigma Aldrich), L-cysteine (Sigma Aldrich), N-ethylmaleimide (NEM, Sigma Aldrich); dithiothreitol (DTT, Sigma Aldrich), fibrillary type I collagen (Helena laboratories, Beaumont, TX) and FITC-conjugated annexin V (BD Bioscience, San Jose, CA). Antibodies used in the study were: a FITC-conjugated monoclonal CD62p (BD Bioscience), a PE-conjugated monoclonal anti-CD42b (BD Bioscience), and antibodies for total and phosphorylated JAK2, STAT3, Syk, and PLCγγ2 (all from Cell Signaling Technologies, Danvers, MA).

### Methods

#### Platelet aggregation

Whole blood was collected (anticoagulant: 3.2% sodium citrate) from healthy donors under protocol #20121746 approved by the Western Institutional Review Board for Bloodworks Northwest via written informed consent. Platelet-rich plasma (PRP) was obtained by centrifugation of whole blood at 120 x g for 20 min at 26°C. Platelet counts in PRP were normalized to 3.0 x 10^5^ platelets/μl with homologous plasma and treated with PL (12.5–100 μM), its synthetic analogs or other testing agents for 15 min at 37°C. Platelet aggregation was induced in an optical aggregometer (Helena Laboratories) by fibrillar type I collagen or a collagen-related peptide (CRP) [22] and monitored for 5 min at 37°C. Collagen was tested primarily at 5 μg/ml in order to test the inhibitory strength of PL activity, which is recommended by the manufacturer for clinical tests, but also tested at 2 and 10 μg/ml in a subset of experiments in order to detect the effects of PL on different concentrations of collagen. PL was also tested for platelet aggregation induced by 5 μM of ADP or 50 μM of thrombin receptor activating peptide (TRAP). To identify the target of PL, the following reagents were tested either alone or in combination with PL at doses indicated in the result section: STA21, AG490, SykII, Actinomycin-D, Apocynin, GSH and L-Cysteine.

#### Platelet activation

The effect of PL on collagen-induced platelet activation was measured by monitoring CD62p expression and calcium influx. CD62 expression was determined through the binding of a FITC-conjugated monoclonal antibody in PRP that was sequentially treated with PL and fibrillar type I collagen (0.5–10 μg/ml), each for 15 min at 37°C. For collagen-induced platelet calcium mobilization, 20 μl of PRP was mixed with 180 μl of Tyrode's buffer (138 mM NaCl, 2.9 mM KCl, 1.4 mM MgCl_2_, 0.8 mM CaCl_2_, 12 mM NaHCO_3_, 5.5 mM glucose, pH 6.5) containing the Calcium Sensor Dye eFluor 514 (5 μM, final concentration, ebioscience, Inc., San Diego, CA). The mixture was immediately analyzed by flow cytometry [23]. Single platelets were gated on forward scatter and analyzed for fluorescence intensity over 60 sec to obtain the basal level. They were then stimulated with collagen, and monitored for changes in fluorescence intensity for additional 5 min at room temperature.

#### Hopping Probe Ion Conductance Microscopy imaging

The effects of PL on the morphological changes and microvesiculation of platelets adherent to collagen were monitored using a modified ICnano Hopping Probe Ion Conductance Microscope (HPICM, Ionscope Ltd, UK) as previously described [24]. Briefly, PL-treated PRP was incubated on immobilized collagen (10 μg/ml, 4°C overnight) for 30 min at 37°C. After washing, adherent platelets were scanned under an ICnano scan head with a nanopipette (d = 60 nm) connected to an external Axon MultiClamp 700B amplifier (Molecular Devices, USA). This amplifier monitored the ion-current flowing into the nanopipette tip and supplied a DC voltage of +200 mV between the nanopipette electrode and bath electrode. All experiments were carried out at 24–28°C. Raw topography data were continuously acquired and linearly interpolated into images with ScanIC Image Viewer software version 1.0 (Ionscope Ltd, UK).

#### Measurement of platelet microparticles

Platelet microvesiculation induced by collagen in the presence or absence of PL was detected by flow cytometry [24]. The supernatant from PRP placed on immobilized collagen (30 min at 37°C) was collected and centrifuged at 16,000x g to remove intact platelets. It was then incubated with a PE-conjugated CD42b antibody and FITC-conjugated annexin V for 20 min at room temperature; and fixed in a HEPES buffered saline containing 2.5 mM of CaCl_2_ and 1% paraformaldehyde. MPs were first identified on particle size gated on forward scatter with a mixture of 0.5, 1.0 and 1.5 μm polystyrene standard beads (BD Bioscience) and then detected for CD42b expression and annexin V binding.

#### PL on thrombus formation

Whole blood samples from healthy subjects were incubated with testing reagents for 20 min at 37°C. They were then perfused over immobilized collagen (coating density: 100 μg/ml, 4°C, overnight) at a flow rate that generated a shear stress of 60 dynes/cm^2^ for 4 min at room temperature in an 8 channel microfluidic chamber (Cellix Mirus Nanopump^™^, Dublin, Ireland). The chamber was then washed with PBS for additional 4 min. Images of thrombi formed on the collagen matrix were recorded at 400 x magnification under an Olympus IX-81 inverted stage microscope.

#### Immunoblots

Washed platelets in Tyrode’s buffer were treated first with a testing agent and then with collagen or/and a mixture of recombinant human IL-6 and the extracellular domain of human IL-6 receptor-α (IL-6-sIL-6α). They were solubilized with a 10X hypotonic lysis buffer (120 mM Tris, 36 mM EDTA, and 10% Triton X-100, pH 7.5) in the presence of a cocktail of protease and phosphatase inhibitors (Sigma Aldrich). Platelet lysates were centrifuged at 13,000 x g for 15 min at 4°C to obtain the supernatant, which was separated on 4–12% gradient SDS polyacrylamide gel electrophoresis (PAGE) and probed for phosphorylated and total JAK2, STAT3, Syk, and PLCγ2 with specific antibodies.

#### Effect of PL on ROS production

Three techniques were used to detect ROS production and its impact on PL activity in collagen treated platelets. First, intracellular ROS was measured by flow cytometry using a cell permeable dye DCFH-DA (Sigma Aldrich), which remains non-fluorescent until the acetate groups are removed by intracellular esterases upon oxidative stress. The dye probe primarily detects HO^**.**^, ONOO-, ROO^**.**^, and H_2_O_2_ species. PRP (150 μl) was diluted in 450 μl of Ca2^+^-free HEPES buffered Tyrode’s (138 mM NaCl, 5.5 mM glucose, 10 mM HEPES, 12 mM NaHCO_3_, 2.9 mM KCl, 0.4 mM NaH_2_PO_4_, 0.4 mM MgCl_2_, and 0.1% BSA, pH 7.2) and incubated with 10 μM of DCFH-DA (final concentration) for 30 min at 37°C. The dye-labeled platelets were analyzed after being treated with 100 μM of PL and/or 10 μg/ml of collagen for 20 min at 37°C. For data validation, the ROS probe CellROX (Life technologies, Grand Island, NY) that detects a similar set of oxidants as DCFH-DA was also tested.

Second, to measure free GSH in platelets, platelets in PRP were normalized to 250,000/μl with platelet-poor plasma and divided into 3 groups, each containing 7.5 x 10^7^ platelets. PRP in two groups were treated with either 100 μM of PL or 10 μg/ml of collagen (final concentrations) for 10 min without stirring. PRP in the third group was left untreated to set a baseline. The treated PRP was incubated with 10 mM of freshly prepared NEM for 60 min at 37°C to block free GSH on and in platelets. The NEM alkylated platelets were quickly frozen at -80°C, thawed on ice and sonicated using a Thermo Fisher Scientific Sonic Dismembrator. The homogenized samples were mixed with isotopically labeled GSH (Sigma Aldrich) internal standard. Proteins were precipitated with methanol and the supernatant was dried by Speed-Vac. Disulfide bonded GSH was reduced by 10mM DTT and newly released free GSH further alkylated by NEM. Total GSH was analyzed using liquid chromatography-tandem mass spectrometry with multiple reaction monitoring (LC-MS/MS-MRM) on an AB Sciex QTrap 6500 mass spectrometer coupled to a Waters Ultra Performance Liquid Chromatographer with Cortex C18 column. The percent of free GSH was calculated by dividing free GSH with total GSH.

Third, to examine a role of ROS production in PL-mediated inhibition, platelets were preincubated with either 20 μM of cell non-permeable GSH or 0.5 mM of cell permeable L-Cysteine for 15 min at 37°C to quench the intracellular and extracellular ROS before being sequentially treated with PL and collagen. The reduced nicotinamide adenine dinucleotide phosphate (NADPH) oxidase inhibitor Apocynin was also similarly tested.

#### PL derivatives

All reactions were carried out under a nitrogen atmosphere in flame-dried glassware except for the hydrogenation reaction. Dichloromethane was purified by passage through activated alumina (solvent purification system). 1H and 13C NMR spectrum were recorded on INOVA-500. 1H NMR chemical shifts are reported as δ values in ppm relative to CDCl3 (7.26 ppm), coupling constants (J) are reported in Hertz (Hz), and multiplicity follows convention. Unless indicated otherwise, deuterochloroform (CDCl3) served as an internal standard (77.2 ppm) for all 13C spectra. Flash column chromatography was performed using 60Å Silica Gel (230–400 mesh) as a stationary phase using a gradient solvent system. Mass spectra were obtained at the center for Chemical Characterization and Analysis (Texas A&M University). Thin layer chromatography (TLC) was performed using silica gel 60 F254 coated glass-back TLC plates.

To generate compound 1 (C1), a mixture of piperlongumine (30 mg, 0.095 mmol) and 10 mg of Pd/C (10 wt.% Pd/C, 0.0094 mmol, 1% equiv) in 10 mL of EtOAc under H2 was stirred at 20°C for 2 h, then was filtered through to a short celite pad. The pad was washed with 10 ml of EtOAc. The solution was concentrated and the crude product was purified by a silica gel flash chromatography (Hexanes:EtOAc = 1:1) to give the desired product as a colorless oil (29 mg, 95%).

Compound 2 (C2) was prepared as a potential probe compound to aid in the identification of the biological target of PL and to provide structure-activity relationships (SAR). C2 has an alkyne group (a latent functional group for further derivatization) that can be used to add additional groups, such as biotin or a fluorophore) that can be used to identify the mode of action of PL. Selective cleavage of the methyl ether at the 4-position of the aromatic ring of PL was achieved using aluminum trichloride in dichloromethane at 0°C to give an intermediate compound in a high yield. Alkylation of the phenol with propargyl bromide produced the alkynyl derivative C2 in 67% yield. Hydrogenation of PL in ethyl acetate gave derivative compound 1 in 95% yield and provided an additional compound for SAR.

#### Statistical analyses

Categorical (frequency) variables were expressed as percentages and continuous variables as mean ± SEM. All quantitative data were analyzed by pair comparisons, one way or repeated measure ANOVA. A p value of less than 0.05 was considered to be statistically significant.

## Results

### Effect of PL on collagen-induced platelet activation and aggregation

**Fig 1:**
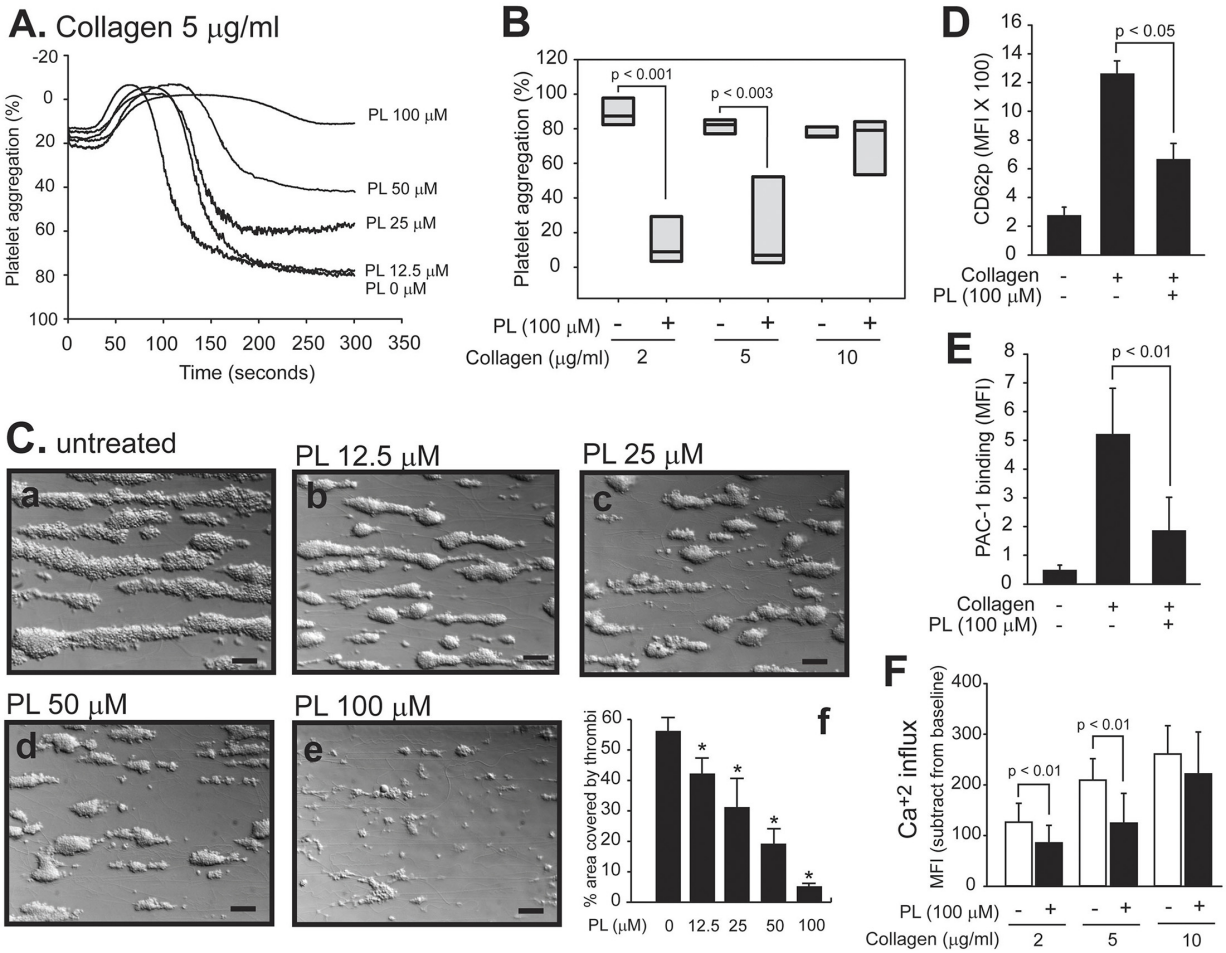
PL inhibited collagen-induced platelet reactivity. (A) Platelets were induced to aggregate by 5 μg/ml of collagen in the presence of increasing concentrations of PL (a representative of 10 separate experiments). (B) PL blocked platelet aggregation induced by low doses, but not a maximal dose of collagen (n = 10, paired *t* test). (C) PL blocked the thrombus formation of platelets on the collagen matrix under flow conditions (panels a-e are representative images [bar = 100 μm] and panel f is a summary of 6 separate experiments, repeated measures ANOVA, * p < 0.01 compared untreated platelets). PL blocked collagen-induced CD62p expression (D), PAC-1 binding (E), and calcium influx (F). Data presented in panels D-F were obtained from 6–8 donors and analyzed by ANOVA (panels D-E) and *t* test (panel F).

**Fig 2:**
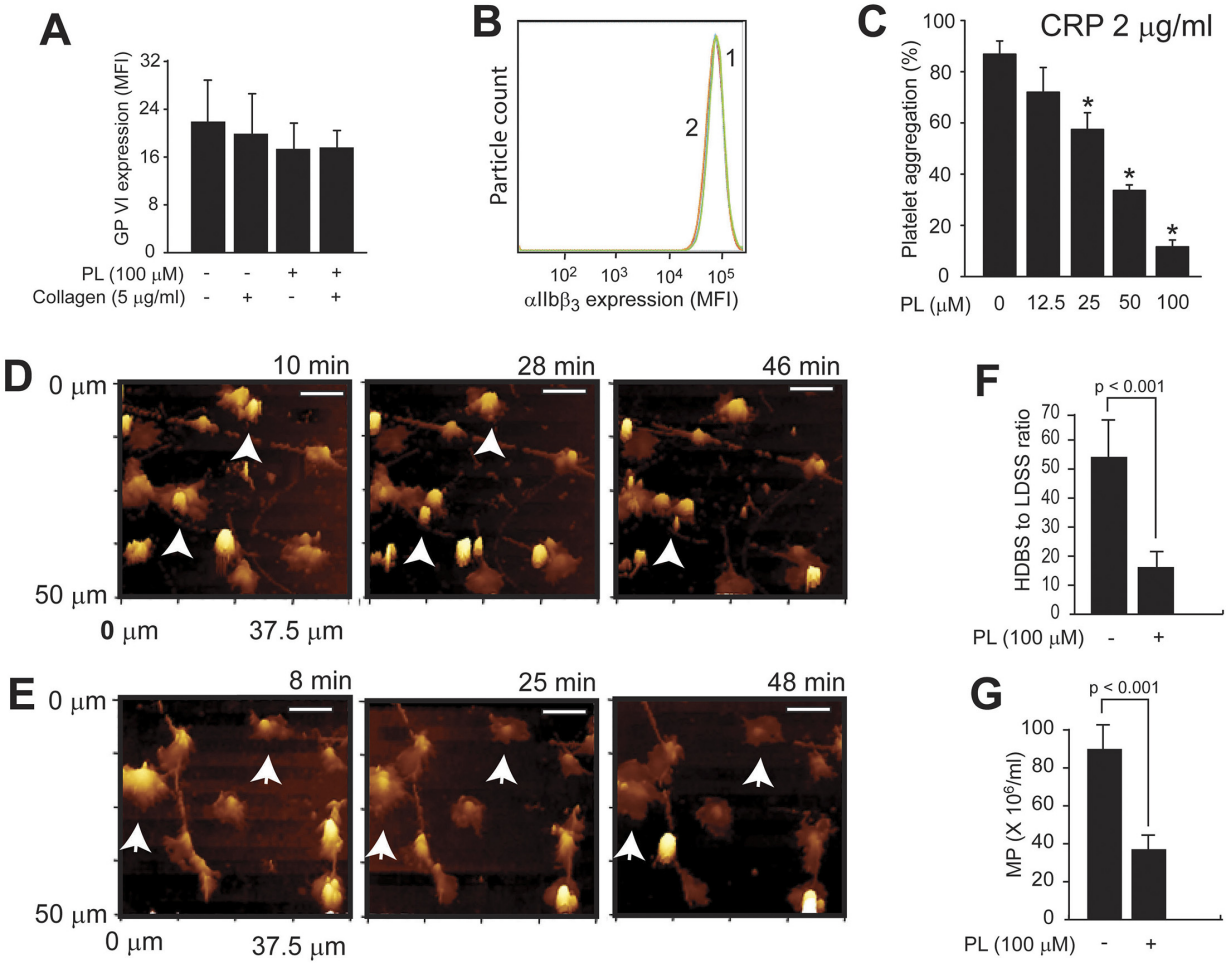
Effects of PL on collagen-induced platelet activation, morphological transition, and microvesiculation. PRP was stimulated with 5 μg/ml of collagen in the presence and absence of 100 μM PL. Surface expressions of GP VI (A, n = 5, ANOVA) and αIIbßß_3_ (B, n = 6, * p < 0.001 compared to PL treated platelets) were measured by flow cytometry using specific monoclonal antibodies. (C) PL dose-dependently blocked CRP-induced platelet aggregation (n = 6, ANOVA). The morphological transition of adherent platelets on the collagen matrix was monitored by HPICM over ~60 min (D & E, bar = 10 μm). A ratio of HDBS to LDSS platelets was calculated (F, n = 6, paired *t* test) and CD42b^+^ and annexin V^+^ platelet microparticles were quantified (G, n = 6, paired *t* test).

PL dose-dependently blocked collagen-induced platelet aggregation with a maximal inhibition achieved at 100 μM (Fig 1A). This inhibitory effect was observed at the subthreshold (2 μg/ml) and high (5 μg/ml) concentration of collagen, but not at a maximal concentration of 10 μg/ml (Fig 1B). PL was equally effective in blocking platelet thrombus formation on immobilized collagen under an arterial shear stress of 60 dynes/cm^2^ (Fig 1C). Consistent with its effect on platelet aggregation and thrombus formation, PL partially blocked the collagen-induced expression of CD62p (Fig 1D), binding of PAC-1, an antibody that specifically recognizes the activated form of the integrin αIIbβ3 (Fig 1E) and calcium influx (Fig 1F). These inhibitory activities of PL were not mediated by inducing receptor shedding because surface densities of GP VI (Fig 2A), which were reduced by collagen stimulation, and the integrin αIIbß_3_ (Fig 2B) were not reduced by the PL treatment. As control, collagen induced a significant increase in the expression of integrin αIIbß_3_. PL also inhibited platelet aggregation induced by CRP (Fig 2C), which specifically targets GP VI, one of two collagen receptors on platelets. In contrast, PL had no effect on collagen-induced aggregation of platelets from mice deficient in platelet STAT3 (S1 Fig). Aggregation of human platelets by ADP (5 μM) and TRAP (50 μM), pretreated with PL also induced aggregation at a level comparable to 5 μg/ml of collagen (S2 Fig).

#### Effect of PL on collagen-induced microparticle production

We previously used a novel HPICM technology to monitor ADP-induced morphological changes and microvesiculation of adherent platelets on immobilized fibrinogen [24]. This technology was modified to monitor real time morphological transformations and microparticle production of platelets on immobilized collagen. As shown in Fig 2D, platelets adherent to collagen presented as predominantly high density bubble shapes (HDBS), which we previously found to be sensitive to agonist-induced microvesiculation [24]. In contrast, many of the PL-treated platelets underwent a reverse transformation from a high-density bubble shape to a low-density spread shape (LDSS, Fig 2E), resulting in a significantly reduced ratio of HDBS to LDSS platelets (Fig 2F) and a reduced production of platelet microparticles (Fig 2G). Together, data presented in Figs 1 and 2 demonstrated an inhibitory activity of PL towards collagen-induced platelet activation and aggregation.

#### Effect of PL on collagen-induced STAT3 activation

**Fig 3:**
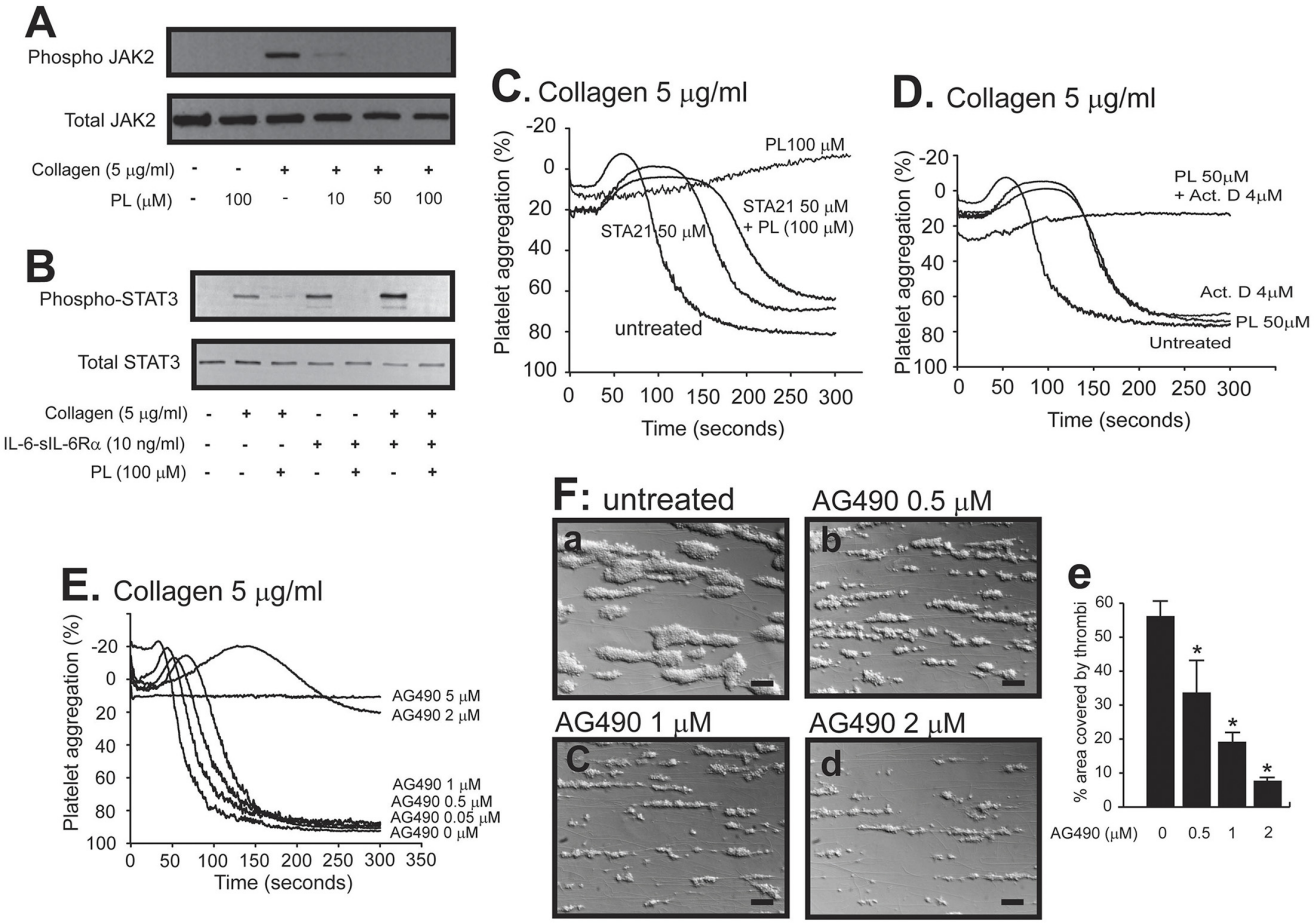
PL blocked JAK2-STAT3 activation. Lysates of platelets stimulated with collagen in the presence of PL were probed for total and phospho-JAK2 (A) and STAT3 (B). STAT3 phosphorylation was also probed in platelets treated with a complex of IL-6-sIL-6α either alone or combined with collagen as control (B). Platelet aggregation was also induced by collagen in the presence of a submaximal 50 μM of PL with and without a maximal inhibitory 50 μM of STA21 (C, a representative of 6 experiments) or actinomycin D (D, a representative of 6 experiments). Platelets treated with an increasing concentration of the JAK2 inhibitor AG490 were induced to aggregate (E) and to form thrombi on immobilized collagen under flow condition (F, bar = 100 μm, the panel e summaries results from 3 experiments, repeated measure ANOVA, *p < 0.01).

We have recently shown that PL potently inhibits STAT3 activation in cancer cells [19] and that STAT3 non-transcriptionally regulates collagen-induced platelet activation/aggregation by facilitating an interaction between the kinase Syk and the substrate PLCγ2 [22]. These results led us to hypothesize that PL targets STAT3 to block collagen-induced platelet reactivity. Here, we provide several lines of experimental evidence to support the hypothesis. First, PL blocked the tyrosine phosphorylation of JAK2 (Fig 3A) and STAT3 (Fig 3B) in platelets stimulated with collagen. As a control, STAT3 phosphorylation induced by a complex of IL-6-sIL-6Rα, which signals through the JAK2-STAT3 pathway in eliciting an acute phase reaction, was also blocked by PL (Fig 3B). Second, PL was significantly less effective in platelets pretreated with the STAT3 inhibitor STA21 (Fig 3C), whereas its effect was additive to that of the transcription inhibitor Actinomycin D (Fig 3D). The latter result suggests that PL acted independent of (mitochondrial) transcription in platelets. Third, the JAK2 inhibitor AG490 dose-dependently blocked collagen-induced platelet aggregation (Fig 3E) and thrombus formation on a collagen matrix under an arterial fluid shear stress of 60 dynes/cm^2^ (Fig 3F).

**Fig 4:**
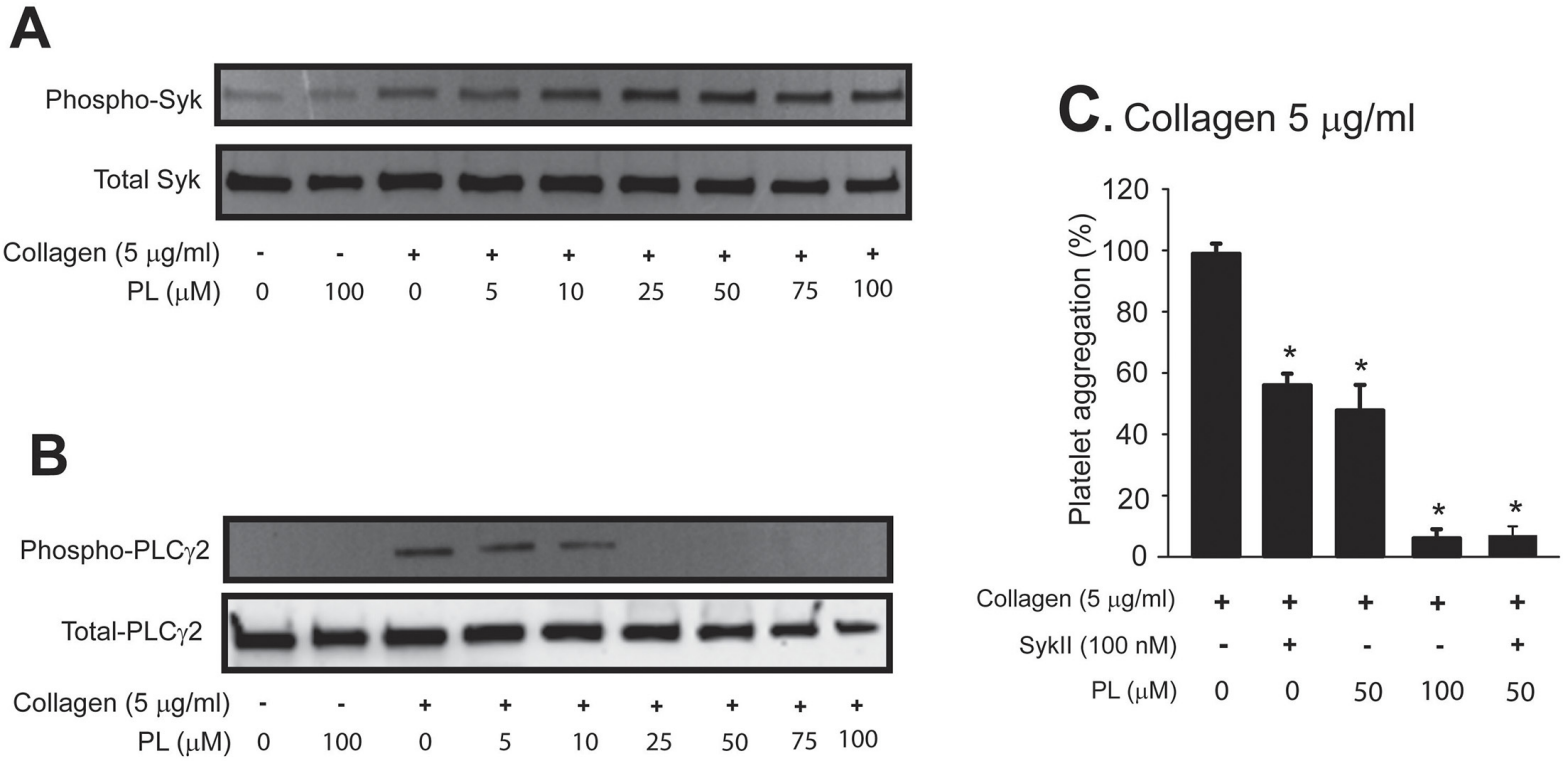
PL effect on collagen-GP VI signaling. Lysates of platelets treated with collagen in the presence of PL were probed for total and phosphorylated Syk (A) and PLCγ2 (B). (C) Platelets were first incubated with SykII for 10 min followed by additional 10 min incubation with PL before being stimulated with collagen (n = 3, repeated measures ANOVA, *p < 0.01).

While PL did not inhibit the tyrosine phosphorylation of Syk (Fig 4A), it was effective in blocking PLCγ2 phosphorylation (Fig 4B) in collagen-stimulated platelets. This regulatory profile of PL is identical to that of STAT3 inhibitors on platelets [22], suggesting that PL does not directly inhibit GP VI-mediated signaling, but disrupts the phosphorylation and subsequent dimerization of STAT3, which are necessary to accelerate or enhance the activity of Syk kinase towards the substrate PLCγ2. Consistent with this notion, the Syk inhibitor SykII inhibited collagen-induced platelet aggregation in synergy with the inhibitory activity of PL (Fig 4C).

#### Differential ROS production in platelets treated with PL or collagen

**Fig 5:**
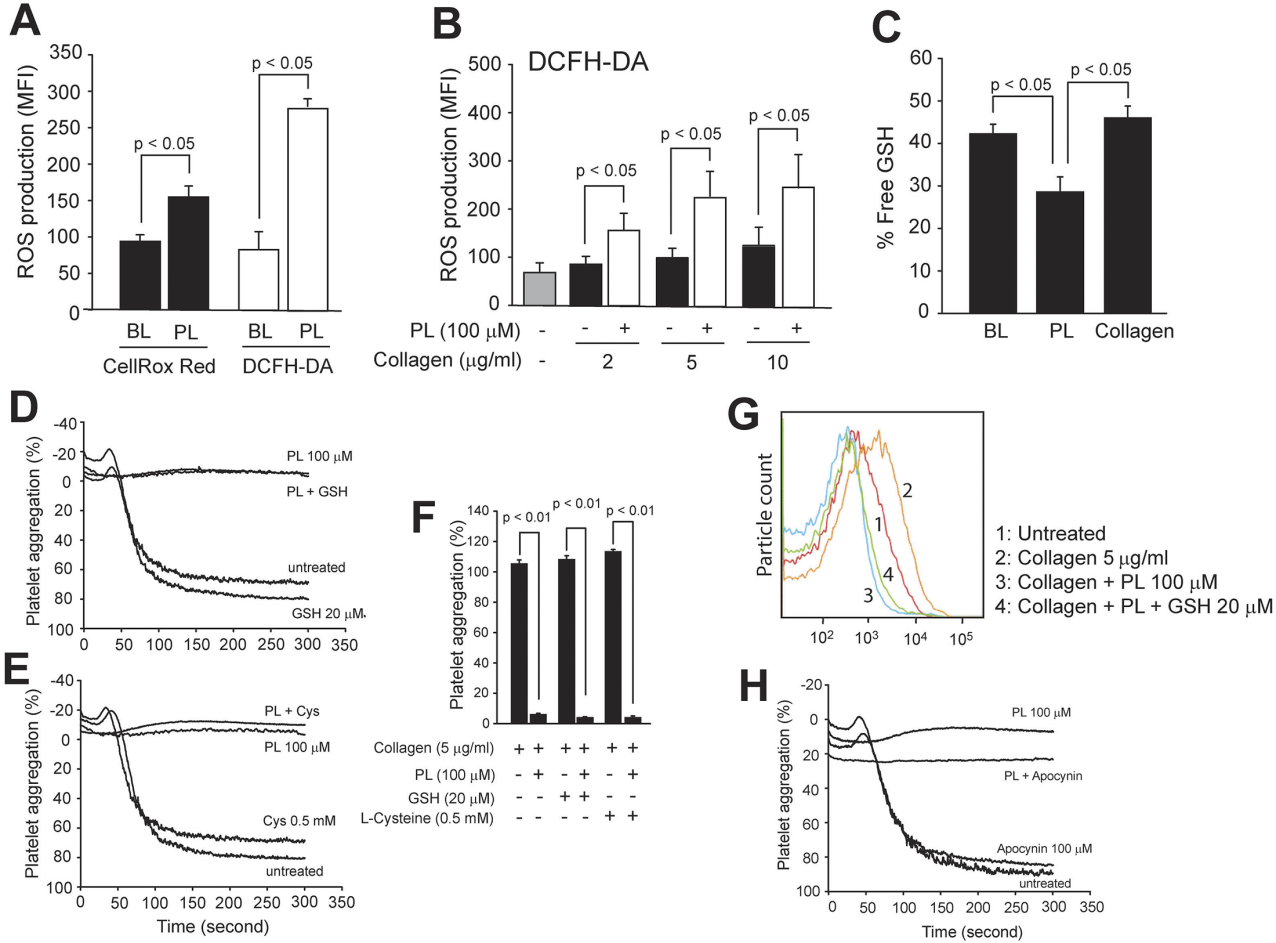
PL-induced ROS production and activity. Platelets in PRP were labeled with either CellROX Red or DCFH-DA for 30 min at 37°C and treated with either100 μM of PL or vehicle control (BL) for additional 10 min. ROS production was measured in platelets by flow cytometry (A, n = 10, paired *t* test). ROS production was also measured in platelets stimulated with various doses of collagen in the presence and absence of PL (B, n = 6, paired *t* test). Free GSH was measured by mass spectrometry in resting platelets and those treated with either PL or collagen for 10 min at 37°C (C, n = 3, one way ANOVA). Collagen-induced aggregation was recorded in platelets preincubated with either GSH (D) or L-cysteine before treatment with 100 μM PL (E). Platelets treated with GSH or L-cysteine without PL were tested as controls (panel F summaries data from 3 experiments). Platelets were treated with collagen, PL, and PL plus GSH for 10 min at 37°C. PAC-1 binding was then detected by flow cytometry (G, a representative of 3 experiments). PRP was incubated with Apocynin for 10 min at 37°C followed by additional 10 min of incubation with 100 μM of PL. Collagen-induced platelet aggregation was then recorded (H, a representative of 3 experiments).

Collagen has been reported to induce ROS release from platelets [18] and several varieties of ROS are known to activate platelets [25], suggesting that ROS is stimulatory to platelets. However, PL has been shown to induce ROS production in cancerous, but not normal cells by binding and down-regulating the activities of GSTP1 and carbonyl reductase 1 CBR1 [11]. These contradictory observations prompted us to examine whether PL induces ROS in platelets. PL at 100 μM that maximally blocked collagen-induced platelet activation and aggregation (Fig 1) induced the release of ROS in platelets as detected by flow cytometry using DCFH-DA and CellRox Red probes (Fig 5A). GSTP1 and CBR1 were also detected in platelets at levels that were not altered by collagen-or/and IL-6-sIL-6α stimulations (S3 Fig). Consistent with previous reports [18], collagen induced platelet production of ROS in a dose dependent manner and its effect was additive to that of PL (Fig 5B). This potent effect of PL on ROS production was further validated by a significant reduction of free GSH in platelets treated with PL, but not with collagen (Fig 5C). However, quenching ROS with 20 μM of cell non-permeable GSH (Fig 5D) or 0.5 mM of cell permeable L-Cysteine (Fig 5E) did not reverse the inhibitory effects of PL on collagen-induced platelet aggregation, even though both anti-oxidants were used at concentrations significantly higher than their baseline plasma levels [26;27]. GSH also failed to reverse PL-induced reduction of PAC-1 binding to collagen-stimulated platelets (Fig 5G). Apocynin, which is an inhibitor of the reduced nicotinamide adenine dinucleotide phosphate (NADPH) oxidase and reported to block collagen-induced ROS production in platelets [18] did not alter the effect of PL on collagen-induced platelet aggregation (Fig 5H).

#### Generation and testing of synthetic analogs

**Fig 6:**
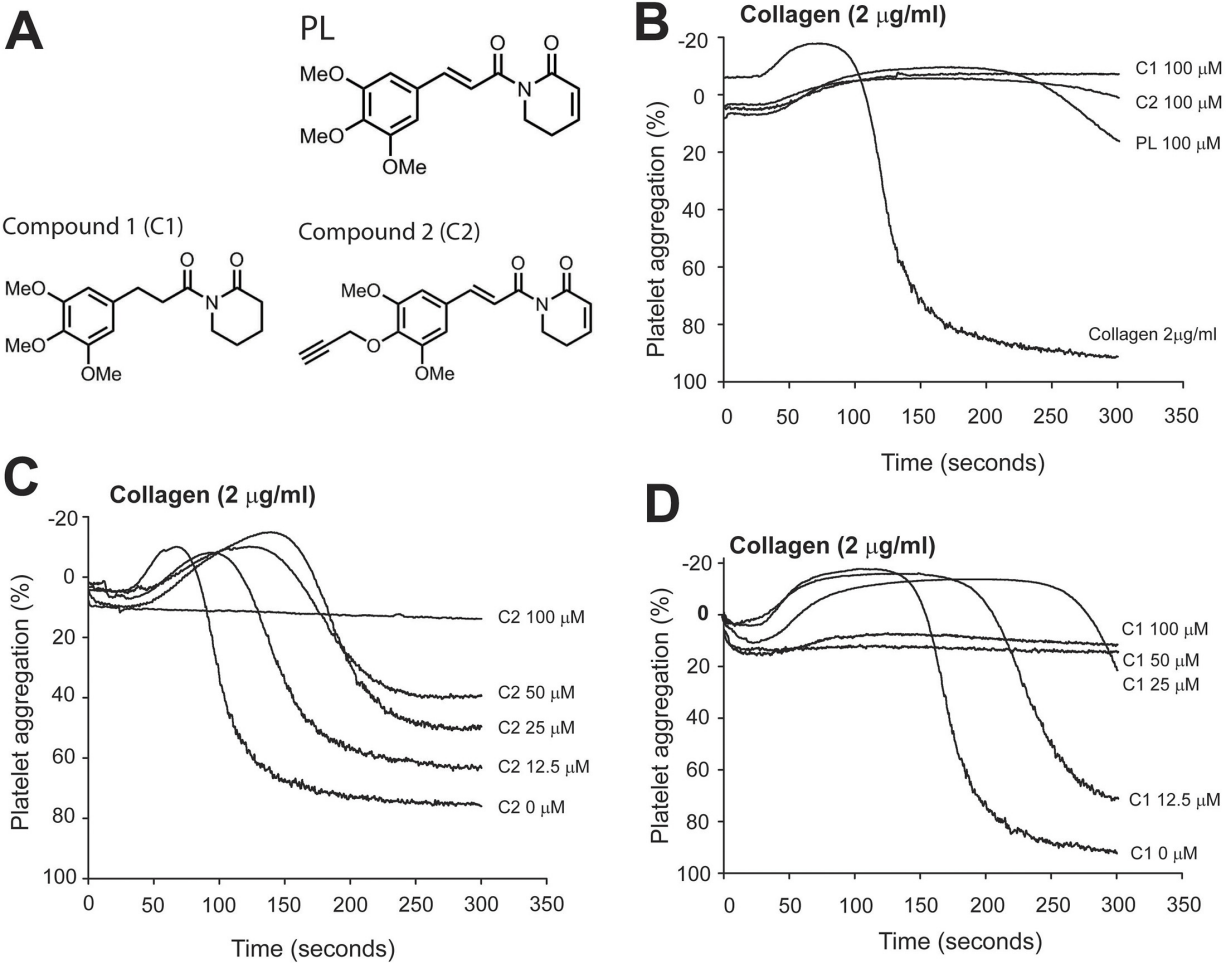
Effects of PL analogs on collagen-induced platelet aggregation. (A) PL and structural derivatives compound 1 (C1) and compound 2 (C2). (B) PRP was incubated with PL, C1 or C2 for 15 min at 37°C and then induced to aggregate by 2 μg/ml of collagen (a representative of 10 experiments). (C & D) C1 and C2 were also tested at different doses for their impact on collagen-induced platelet aggregation.

The diverse effects of PL on cancer cells and platelets may result from different structures and chemical moieties. To examine the role of specific chemical moieties in blocking platelet reactivity to collagen, we synthesized two PL analogs (Fig 6A) through hydrogeneration [3] and by introducing a terminal alkyne [11] at a site away from the possible “bioactivity bearing” functional groups (compound 1 & 2). The two derivatives were found to be active in blocking platelet aggregation induced by 2 μg/ml (Fig 6B and 6D), but not at 5 μg/ml of collagen (data not shown).

## Discussion

PL has been reported to be inhibitory to several major platelet agonists. Here, we have specifically characterized inhibitory effects of PL on collagen-induced platelet reactivity (Figs 1 and 2) to explore the influence of PL on the platelet JAK2- STAT3 pathway. Our findings are consistent with previous reports [13;14]. We have also made several new observations that define the mechanism by which PL inhibits collagen-induced platelet activation and provide new information regarding the differential effects of ROS on platelet reactivity to collagen.

First, PL inhibited collagen-induced platelet reactivity primarily by blocking the activation of JAK2 and STAT3. This conclusion is supported by several lines of experimental evidence presented in Figs 3 & 4. In nucleated cells, tyrosine phosphorylated STAT3 dimerizes to be more efficiently translocated into the nucleus to regulate the transcription of multiple genes associated with the acute phase reaction [28–31]. We have recently shown that a STAT3 dimer enhances collagen-induced signaling in platelets by serving as a protein scaffold for Syk and PLCγ2 [22] and PL prevents this critical dimerization by preventing JAK2-mediated STAT2 activation [19]. Furthermore, PL did not directly inhibit collagen-induced Syk phosphorylation, but did block the activation of its substrate PLCγ2. This regulatory profile is consistent with the notion that a phosphorylated STAT3 dimer serving as a protein scaffold linking the kinase Syk to the substrate PLCγ2 in the GP IV signal pathway. Furthermore, the JAK2 inhibitor AG-490 (Tyrphostin B42), which has no effect on the kinases Lyn, Syk and Src involved in GP VI signaling, also blocked collagen-induced platelet aggregation and thrombus formation under flow conditions (Fig 3E and 3F). Together, these data suggest that PL directly prevents STAT3 dimerization and indirectly by blocking JAK2 activation.

Second, both PL and collagen induced ROS production in platelets (Fig 5A–5C), but PL’s inhibitory activity on collagen-induced platelet activation was not affected by the ROS (Fig 5D–5G). The results raise two interesting questions. The first regards the previous reports that PL induces ROS production by inhibiting GSTP1 activity specifically in cancerous, but not normal cells, leading to ROS-mediated apoptosis [3;11]. Platelets treated with PL did not undergo apoptosis as they remained aggregatable in response to high doses of collagen (Fig 1B), ADP (S1 Fig) and TRAP (S1 Fig). They also had a reduced rate of microvesiculation (Fig 2E–2H). The second question regards the impact of ROS on platelet reactivity. The inhibitory effects of PL on collagen-induced platelet reactivity was not affected by cell permeable and non-permeable reducing agents that quench extracellular and intracellular oxidants; and by a NADPH oxidase inhibitor (Fig 5D–5G), which blocked collagen-and thrombin-induced ROS production in platelets [17;18]. The finding strongly suggests that PL and collagen induce the release of different oxidants that have differential effects on platelets. For example, PL induces cancer cells to produce nitric oxide [11], which is a well-known platelet inhibitor [32]. Our finding also differs from the reported effect of the GSH precursor N-acetyl-L-cysteine on eliminating PL’s effect on cancer cells [11], implying that PL acts differently in cancer cells and in platelets. Studying these differential effects on drug-induced oxidative stress may uncover new mechanisms of regulating platelet functions by selective oxidants.

Third, we made and tested two PL analogs with specific structure modifications. These analogs are designed to help differentiate the contributions of specific structures on inhibitory activity of PL in collagen-induced platelet reactivity. The double bonds C3-C4 and C7-C8 in PL were reduced in compound 1 to make the analog less reactive. It has also been shown that the electrophilicity of the C2-C3 olefin is critical for the apoptotic effects of PL on cancer cells, whereas analogs lacking a reactive C7-C8 olefin induce intracellular ROS, but markedly reduced cell death [3]. In contrast, compound 2 was designed as a potential probe to identify biological targets of PL and to provide structure-activity relationships. However, both derivatives are equally active in blocking platelet reactivity to the low dose of collagen (Fig 6).

In summary, we have shown that PL inhibits collagen-induced platelet activation, aggregation, and thrombus formation by primarily blocking JAK2-STAT3 phosphorylation, reducing the ability of STAT3 to serve as a protein scaffold for linking Syk to PLCγ2. We further show that, while PL induces ROS in platelets, its inhibitory activity towards collagen-induced platelet activation was not affected by ROS, suggesting that ROS have differential effects on platelet reactivity.

## Supporting Information

S1 Fig Citrated PRP from STAT3^-/-^ and littermate control mice were preincubated with either PL and the vehicle DMSO for 10 min at 37°C and then stimulated with 0.75 μg/ml of fibrillary type I collagen. Platelet aggregation was monitored for 10 min at 37°C. We have previously shown that, at this dose of collagen, the collagen-induced aggregation of platelet from STAT3^-/-^ was significantly reduced as compared to control platelets (22). Here, we further show that PL was not active in blocking collagen-induced aggregation of platelets from the STAT3^-/-^ mice (n = 8 mice/group, paired t test).
(JPG)

S2 Fig Platelet aggregation induced by 5 μM of ADP (A) or 50 μM of TRAP (B) was not blocked by PL. (JPG)

S3 Fig Expression of GSTP-1 and CBR-1 in platelets detected by immunoblots. (JPG)
