# Correction: Piperlongumine Blocks JAK2-STAT3 to Inhibit Collagen-Induced Platelet Reactivity Independent of Reactive Oxygen Species^†^

**DOI:** 10.1371/journal.pone.0146626

**Published:** 2016-01-04

**Authors:** Hengjie Yuan, Katie L. Houck, Ye Tian, Uddalak Bharadwaj, Ken Hull, Zhou Zhou, Mingzhao Zhu, Xiaoping Wu, David J. Tweardy, Daniel Romo, Xiaoyun Fu, Yanjun Zhang, Jianning Zhang, Jing-fei Dong

The seventh author’s name is incorrect. The correct name is: Mingzhao Zhu.
