# Supplementary figures and images for: Piperlongumine Blocks JAK2-STAT3 to Inhibit Collagen-Induced Platelet Reactivity Independent of Reactive Oxygen Species†
Source: PLoS One. 2015 Dec 8;10(12):e0143964. doi: 10.1371/journal.pone.0143964 (PMC4672935; doi:10.1371/journal.pone.0143964)

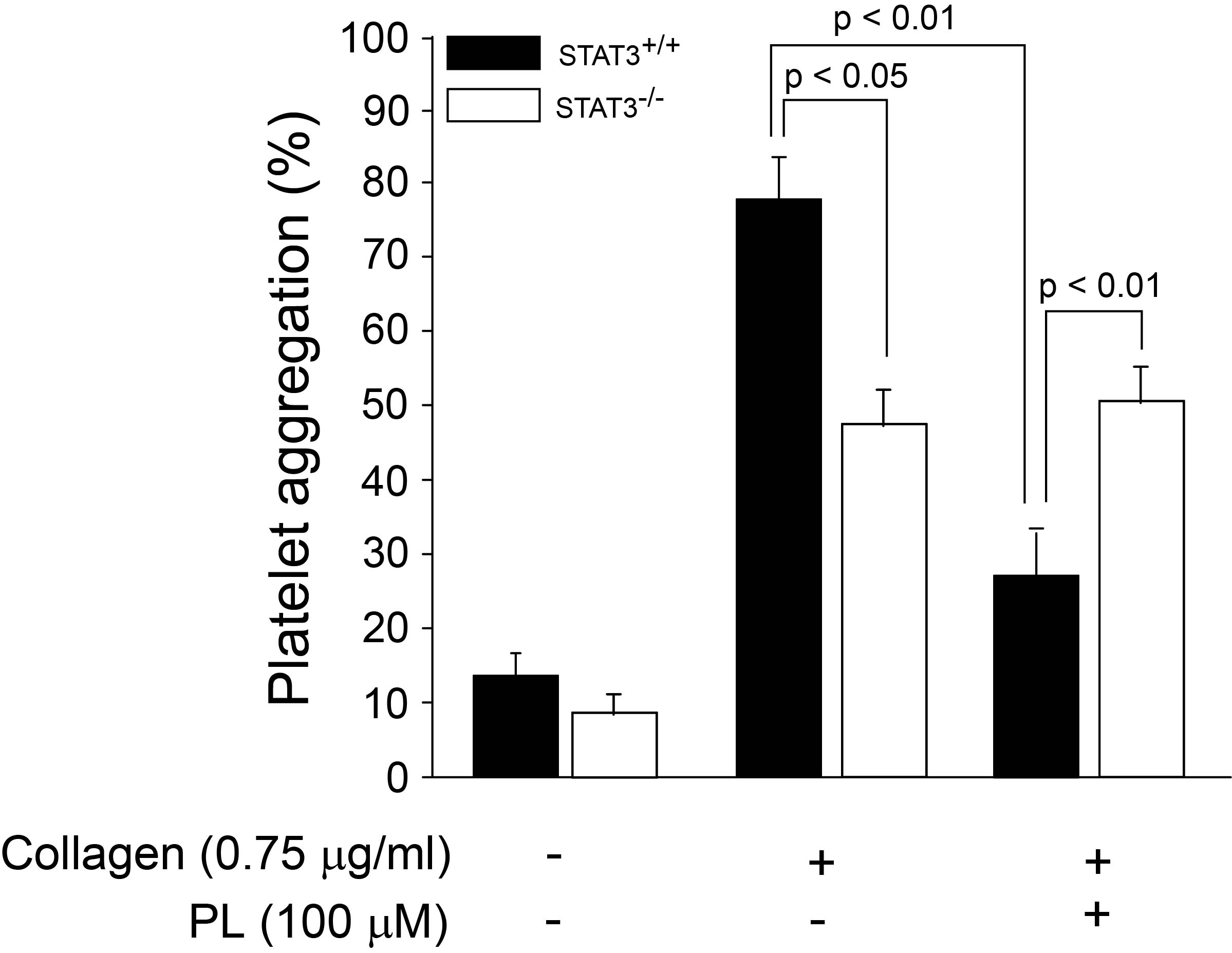

Supplement: S1 Fig — Platelet aggregation was monitored for 10 min at 37°C. We have previously shown that, at this dose of collagen, the collagen-induced aggregation of platelet from STAT3-/- was significantly reduced as compared to control platelets (22). Here, we further show that PL was not active in blocking collagen-induced aggregation of platelets from the STAT3-/- mice (n = 8 mice/group, paired t test). (JPG) [file pone.0143964.s001.jpg]

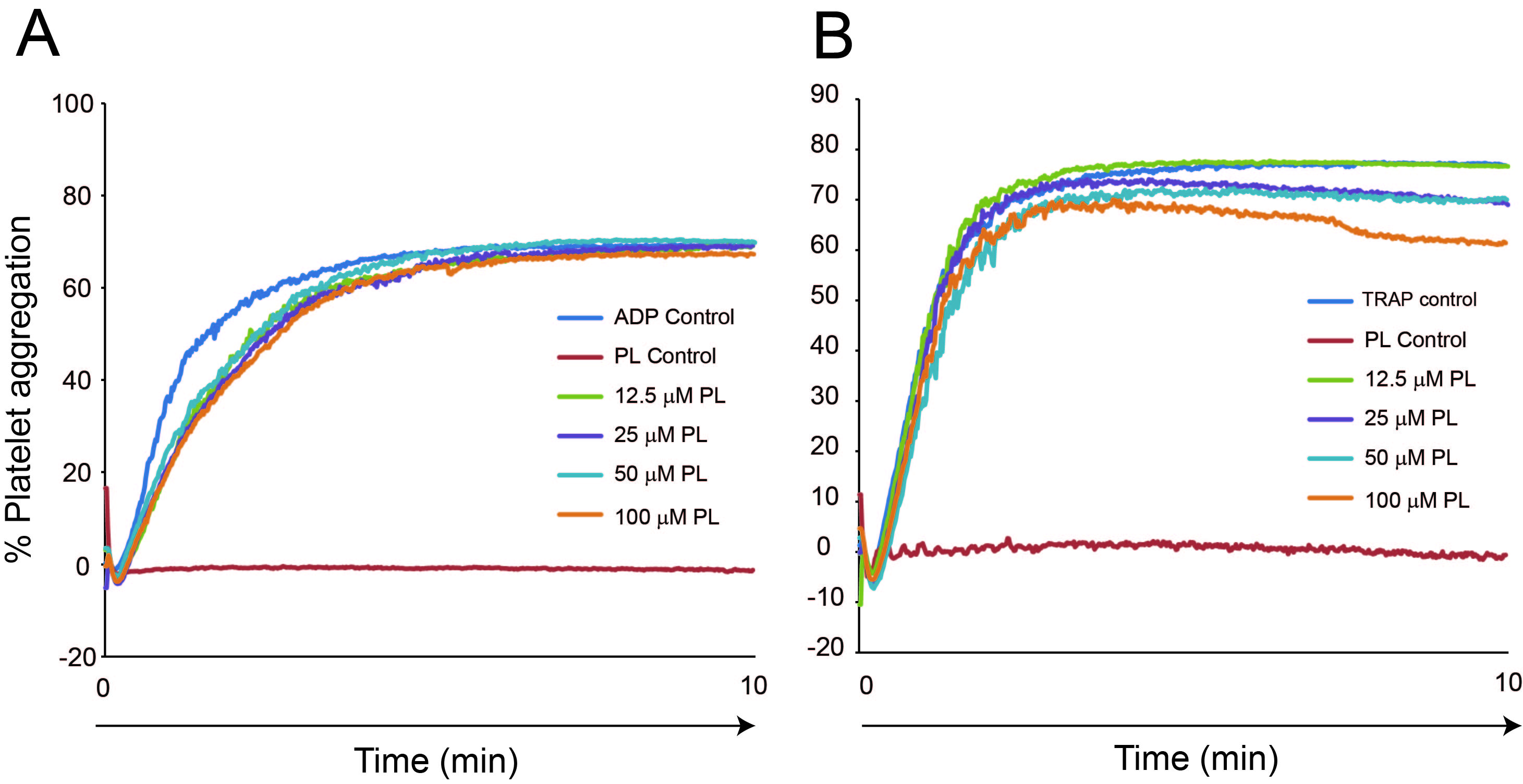

Supplement: S2 Fig — (JPG) [file pone.0143964.s002.jpg]

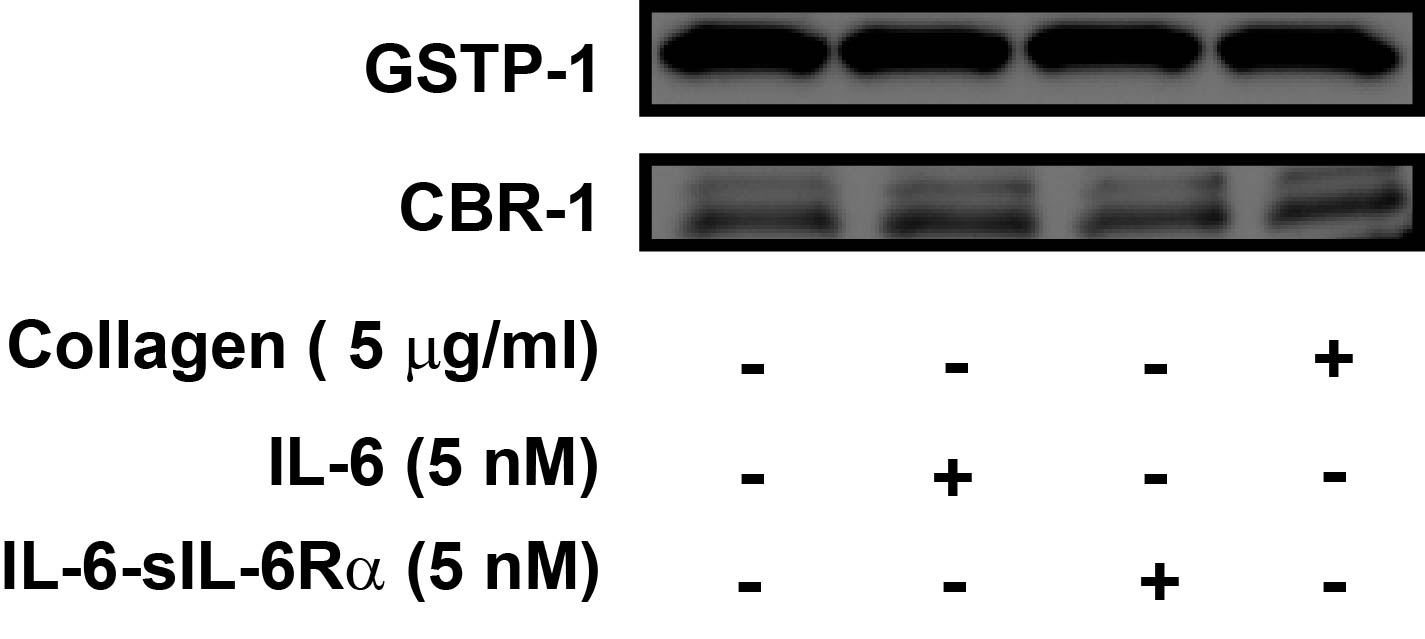

Supplement: S3 Fig — (JPG) [file pone.0143964.s003.jpg]
